# Supplementary material for: Paeoniflorin Enhances Endometrial Receptivity through Leukemia Inhibitory Factor
Source: Biomolecules. 2021 Mar 16;11(3):439. doi: 10.3390/biom11030439 (PMC8002267; doi:10.3390/biom11030439)
Supplement: Supplementary file 1 [file biomolecules-11-00439-s001.zip › Supplementary Figures_R1.docx]

**Supplementary Figures**

**
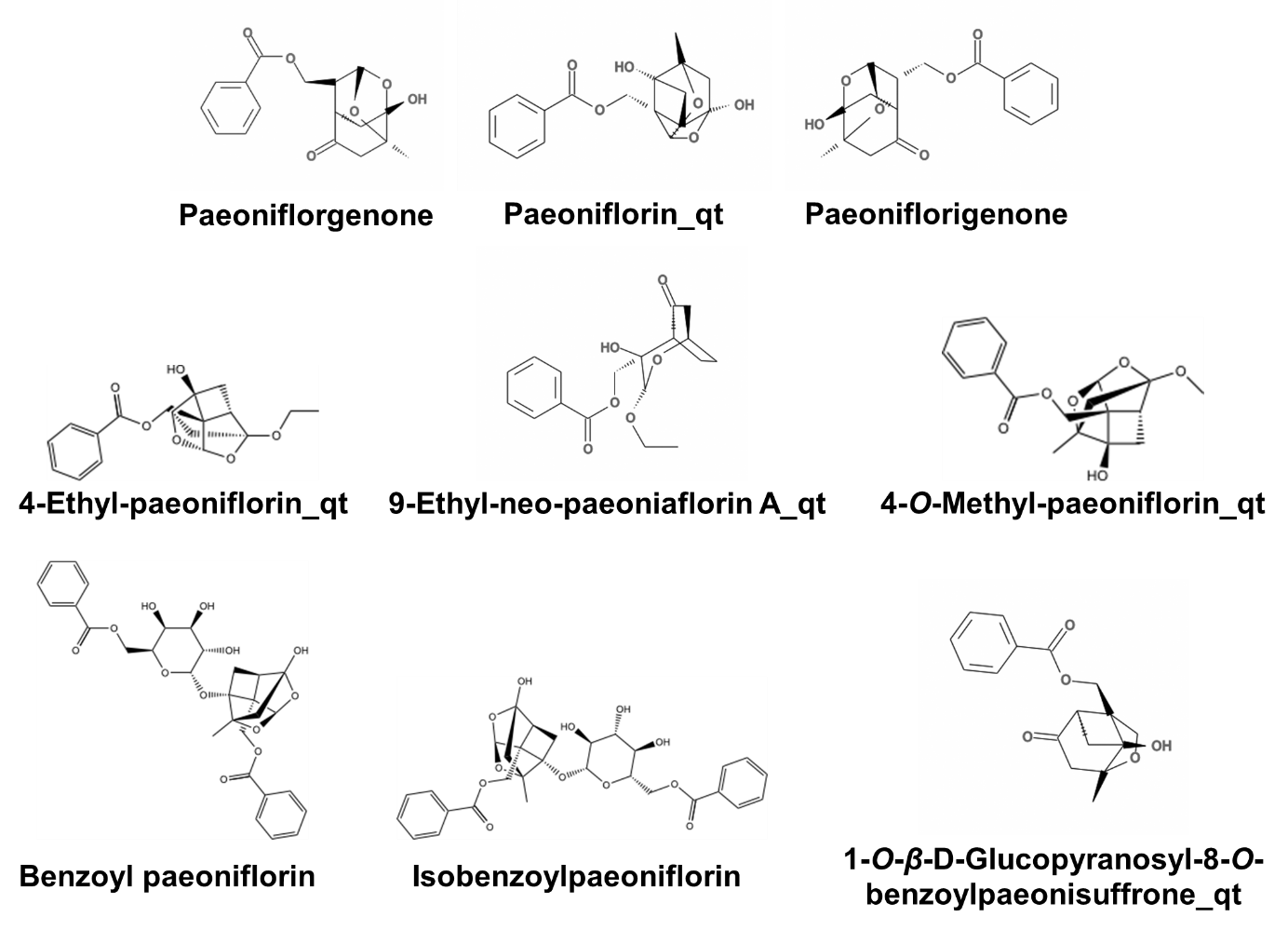
**

**Figure S1. Structures of paeoniflorin derivatives in screened compound list of *Paeonia lactiflora* obtained from Traditional Chinese Medicine Systems Pharmacology (TCMSP) analysis**


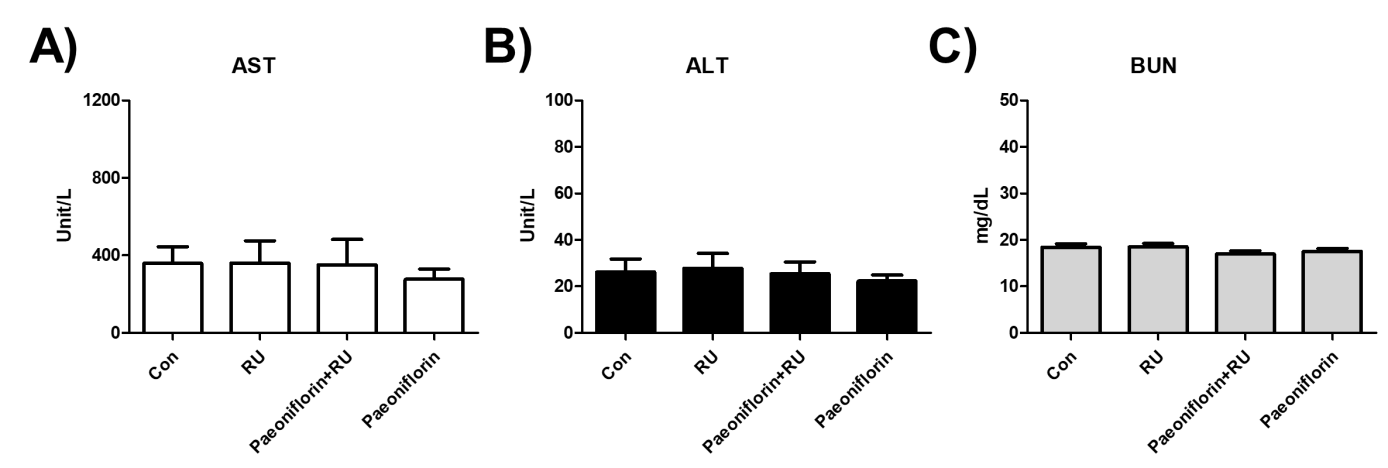


**Figure S2. Effect of paeoniflorin on liver and kidney toxicity.** Sera were collected from female mice after embryo implantation experiment. Possible toxicity of paeoniflorin on liver and kidney were screened using commercially available aspartate transaminase (AST), alanine transaminase (ALT), and blood urea nitrogen (BUN) analysis, serviced by Greencross (Seoul, Korea).


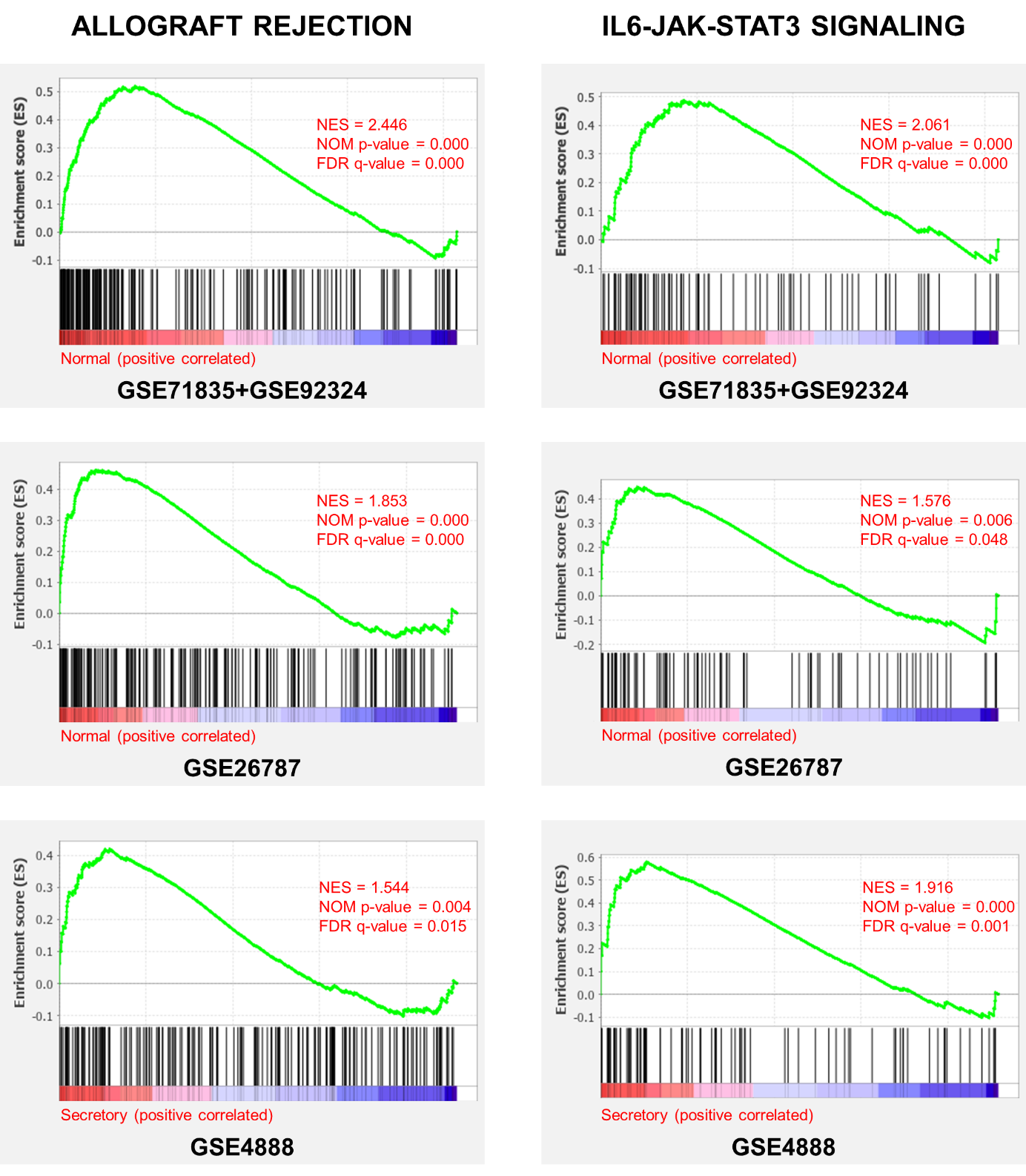


**Figure S3. Gene set enrichment analysis (GSEA) enrichment plots of allograft rejection and interleukin-6/Janus kinase/signal transducer and activator of transcription 3 (IL6/JAK/STAT3) signaling gene sets.** High normalized enrichment score (NES) value indicates gene set is overrepresented at top of ranked gene list in normal (GSE71835+GSE92324 and GSE26787) or secretory phase (GSE4888) human endometrial transcriptome. Statistical significance was determined using NOM (Nominal) p-value<0.05 and false discovery rate (FDR) q-value<0.25.
