# Supplementary material for: Paeoniflorin Enhances Endometrial Receptivity through Leukemia Inhibitory Factor
Source: Biomolecules. 2021 Mar 16;11(3):439. doi: 10.3390/biom11030439 (PMC8002267; doi:10.3390/biom11030439)
Supplement: Supplementary file 1 [file biomolecules-11-00439-s001.zip › Table S1. The pharmacokinetic parameters of gallic acid, methyl gallate, and paeonol.docx]

**Table S1. Pharmacokinetic (PK) parameters of gallic acid, methyl gallate, and paeonol analyzed by Traditional Chinese Medicine Systems Pharmacology (TCMSP)**

**
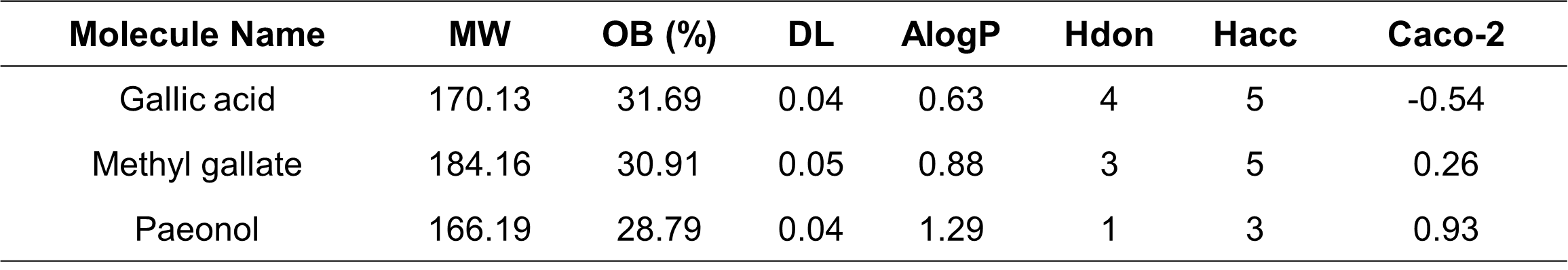
**

These three components of *Paeonia lactiflora* did not satisfy screening criteria (oral bioavailability [OB] ≥ 30% and drug likeness [DL] ≥ 0.18)

Note: MW, molecular weight; OB, oral bioavailability; DL, drug likeness; AlogP, octanol-water partition coefficient log P; Hdon, hydrogen donor; Hacc, hydrogen bond acceptor; Caco-2, Caco-2 permeability.
